# LncRNA KCNQ1OT1 activated by c-Myc promotes cell proliferation via interacting with FUS to stabilize MAP3K1 in acute promyelocytic leukemia

**DOI:** 10.1038/s41419-021-04080-1

**Published:** 2021-08-17

**Authors:** Doudou Tang, Yujiao Luo, Yafeng Jiang, Piao Hu, Hongling Peng, Shangjie Wu, Guangsen Zhang, Yewei Wang

**Affiliations:** 1 Department of Respiratory and Critical Care Medicine, the Second Xiangya Hospital, Central South University, Changsha, Hunan China; 2 Hunan Centre for Evidence-based Medicine, Central South University, Changsha, Hunan China; 3 Department of Hematology, The Second Xiangya Hospital, Central South University, Changsha, Hunan China; 4 Institute of Molecular Hematology, Central South University, Changsha, Hunan China

**Keywords:** Acute myeloid leukaemia, Acute myeloid leukaemia

## Abstract

Uncontrolled proliferation is the hallmark of cancer cells. Previous studies mainly focused on the role of protein-coding genes in cancer cell proliferation. Emerging evidence showed that long non-coding RNAs (lncRNAs) also play critical roles in cancer cell proliferation and growth. LncRNA KCNQ1OT1 is found to contribute to carcinogenesis, but its role in acute promyelocytic leukemia (APL) is unclear. In this study, by analyzing data from Gene Expression Omnibus, The Cancer Genome Atlas database and our clinical samples, we found that KCNQ1OT1 was selectively highly expressed in APL. Functional assays demonstrated that knockdown of KCNQ1OT1 reduced APL cell proliferation and increased apoptosis. Further evidence showed that KCNQ1OT1 was mainly located in the cytoplasm of APL patient-derived NB4 cells and APL patient bone marrow samples. Mechanistically, KCNQ1OT1 bound to RNA binding protein FUS, and silencing either KCNQ1OT1 or FUS reduced the expression level and stability of MAP3K1 mRNA. Whereas KCNQ1OT1 and FUS did not affect each other. Importantly, knockdown of MAP3K1 impaired APL cell proliferation. Finally, c-Myc transactivated KCNQ1OT1 in APL cells through binding to its promoter while knockdown of c-Myc decreased KCNQ1OT1 expression. Our results not only revealed that c-Myc transactivated KCNQ1OT1 and upregulated KCNQ1OT1 promoted APL cell proliferation, but also demonstrated that KCNQ1OT1 bound to FUS to synergistically stabilize MAP3K1 mRNA, thus facilitating APL cell proliferation. This study established a previously unidentified role of KCNQ1OT1 in the development of APL, and KCNQ1OT1 may serve as a potential therapeutic target for APL.

## Introduction

Uncontrolled proliferation, which is the outcome of a complex multifactorial process, is the hallmark of cancer cells [1]. Previous studies mainly focused on the function of protein-coding genes in cancer cell proliferation. Recently, long non-coding RNAs (lncRNAs), which can be transcribed from a considerable fraction of the human genome [2], have been found to play an essential roles in cancer cell proliferation [3]. However, lncRNAs reported to be involved in leukemia cell proliferation are limited. Identifying functional lncRNAs critical for leukemia cell proliferation and growth will provide comprehensive insights into the pathogenesis of leukemia and new potential targets for leukemia treatment.

Acute promyelocytic leukemia (APL), the M3 subtype of acute myeloid leukemia (AML), is characterized by hyperproliferation of leukemic promyelocytes in the bone marrow and/or peripheral blood. APL typically presents with a life-threatening hemorrhagic disorder and is a highly lethal disease historically [4, 5]. Application of all-*trans* retinoic acid and arsenic trioxide makes APL a highly curable disease during the past three decades [6, 7]. Therefore, a full understanding of the pathogenesis of APL will have an important implication for underlying mechanisms and clinical treatments of other subtypes of AML.

KCNQ1OT1, a 91 kb-long non-protein-coding antisense transcript, is initially discovered to be responsible for transcriptional silencing of genes in the KCNQ1 cluster. This cluster is an important tumor suppressor gene region [8]. The mechanistic investigation found that in the nucleus, KCNQ1OT1 could interact with DNMT1, histone methyltransferases G9a and the PRC2 complex to silence the expression of genes within the parental region, including the CDKN1C gene, which encodes cell cycle inhibitor p57 [9, 10]. Clinically, overexpression of KCNQ1OT1 is frequently reported in Beckwith-Wiedemann syndrome patients, and about 10% of these patients developed embryonal tumors [11], suggesting its pro-oncogenic potential. Consistently, growing evidence has shown the crucial roles of KCNQ1OT1 in the initiation and invasion of cancers [12–14]. For example, KCNQ1OT1 was highly expressed in lung adenocarcinoma and high expression of KCNQ1OT1 is correlated to malignant behaviors, including large tumor size, higher lymph nodes metastasis rate, and advanced TNM stage. Whereas silencing of KCNQ1OT1 represses cell proliferation and invasion [15]. In glioma, KCNQ1OT1 was upregulated and KCNQ1OT1 upregulation promotes tumor cell proliferation through activating miR-370/CCNE2 axis [16]. In addition, KCNQ1OT1 was also upregulated in colorectal cancer (CRC), which accelerates the proliferation, migration, and epithelial–mesenchymal transition (EMT) of CRC cells via regulating miR-217/ZEB1 axis [17]. These results suggest that KCNQ1OT1 contributes to carcinogenesis through the competing endogenous RNA (ceRNA) mechanism in the cytoplasm. However, the expression and role of KCNQ1OT1 in APL remain unclear.

In the present study, we first analyzed data from Gene Expression Omnibus (GEO), The Cancer Genome Atlas (TCGA) database and our clinical samples, and found that KCNQ1OT1 was selectively highly expressed in APL. Functional assays demonstrated that knockdown of KCNQ1OT1 reduced APL cell proliferation. Mechanistically, KCNQ1OT1 bound to RNA binding protein FUS to form a complex, which stabilized MAP3K1 mRNA. Moreover, c-Myc transactivated KCNQ1OT1 in APL cells through binding to its promoter. Our results emphasize an oncogenic role of KCNQ1OT1 in the development of APL.

## Materials and methods

### Bioinformatics analysis

The expression profiles of KCNQ1OT1 in human tumor samples and paired normal tissues were obtained from the GEPIA website (http://gepia.cancer-pku.cn). The correlations between KCNQ1OT1/FUS and MAP3K1 in AML were also obtained from the GEPIA website. The microarray gene expression data were downloaded from GEO with accession numbers GSE10358 and GSE12662. The data of RNA-sequencing in AML were downloaded from the TCGA database (https://cancergenome.nih.gov/). The potential interactions of FUS with KCNQ1OT1 and MAP3K1 were predicted by ENCORI (http://starbase.sysu.edu.cn/index.php) and RBPmap (http://rbpmap.technion.ac.il). The interaction scores between KCNQ1OT1/MAP3K1 mRNA and FUS were also predicted by the RNA-Protein interaction prediction (RPISeq) website (http://pridb.gdcb.iastate.edu/RPISeq/). The binding sites on the KCNQ1OT1 promoter for c-Myc were predicted by the JASPAR tool (http://jaspar.genereg.net/).

### Patient samples

Bone marrow samples were obtained from 19 patients with de novo APL and eight cases with normal bone marrows (health and non-leukemia patients). Leukemic cells isolated from bone marrows with >90% blasts were cultured as previously described [18] and used for lentiviral transfection. Patients characteristics were summarized in Supplementary Material: Table S1.

### Cell culture and reagent

NB4 cells were grown in RPMI 1640 (Gibco, Carlsbad, CA, USA) containing 10% fetal bovine serum (FBS) (Gibco). The 293T cells were cultured in DMEM (Gibco) supplemented with 10% FBS. Cells were incubated in a humidified atmosphere with 5% CO_2_ at 37 °C.

### Quantitative real-time RT-PCR

Total RNA was extracted with RNAiso plus (TaKaRa, Dalian, Liaoning, China) and reverse transcription was conducted with PrimeScript RT reagent Kit (TaKaRa). Quantitative real-time PCR (qRT-PCR) was performed on the Roche LightCycler 96 system using the SYBR Premix Ex Taq II (TaKaRa). GAPDH was used for normalization. All primers for quantitative real-time RT-PCR are listed in Supplementary Material: Table S2.

### Subcellular fractionation location

The separation of nuclear and cytoplasmic fractions was performed using the PARIS Kit (Thermo Fisher Scientific, Carlsbad, CA, USA) according to the manufacturer’s instructions. qRT-PCR was used to determine the RNA (KCNQ1OT1, GAPDH, and U6) levels in fractions.

### Cell proliferation assay

NB4 cells transfected with shKCNQ1OT1 or shMAP3K1 were seeded in 96-well plates at a density of 1 × 10^5^ cells/ml. Cell proliferation was quantified by the Cell Counting Kit-8 (CCK-8; Dojindo, Kumamoto, Japan) every 24 h following the manufacturer’s instructions.

### Colony formation assay

To assess the colony-forming efficiency of NB4 cells, transfected cells were plated at a concentration of 5 × 10^2^ cells/ml in RPMI 1640 supplemented with methylcellulose and 10% FBS. After incubated for 2 weeks, cells were fixed with 4% paraformaldehyde and stained with crystal violet (Sigma-Aldrich). The visible colonies were counted manually and photographed microscopically.

### Flow cytometry

For cell cycle analysis, transfected cells were collected and fixed with 1% formaldehyde at 4 °C for 1 h. After washed with PBS, cells were permeabilized with 70% ethanol overnight at −20 °C. Then cells were treated with 100 μg/ml RNase A at 37 °C for 30 min. Subsequently, cells were stained with PI for 30 min. GFP-positive cells were analyzed for DNA content by a BD FACS Canto II flow cytometer (BD Biosciences).

For cell apoptosis analysis, cells were double stained with APC-Annexin V and Propidium iodide (PI) by using the APC-Annexin V Apoptosis Detection Kit with PI (BioLegend, San Diego, CA, USA) according to the manufacturer’s instructions. Then GFP-positive cells were analyzed by flow cytometry.

### Western blot

Cell protein lysates were subjected to SDS-polyacrylamide gel electrophoresis. Separated proteins were transferred to PVDF membranes and treated with 5% skimmed milk, then incubated with corresponding primary antibodies against MAP3K1 (Proteintech, 19970-1-AP), FUS (Abcam, ab124923), and GAPDH (Proteintech, 10494-1-AP) overnight at 4 °C followed by HRP-conjugated secondary antibodies for 2 h. Band density was analyzed using an ECL kit (Invitrogen, Carlsbad, CA, USA).

### Chromatin immunoprecipitation (ChIP) assay

ChIP was performed using Pierce Agarose ChIP Kit (Thermo Fisher Scientific, Rockford, IL, USA) according to the manufacturer’s instructions. The following antibodies were used: c-Myc (Santa Cruz Biotech, sc-42x) and rabbit IgG (Abcam, ab46540). Immunoprecipitated DNA was analyzed by qPCR. All primers for ChIP-qPCR are listed in Supplementary Material: Table S2.

### RNA interference experiments and transfection

Lentiviral plasmids expressing short hairpins against KCNQ1OT1/MAP3K1/FUS/c-Myc and negative control were constructed using pLVX-shRNA2 vector (Clontech Laboratories, Mountainview, CA, USA) following the manufacturer’s instructions. Lentiviral particles were generated by co-transfecting 293T cells of lentiviral plasmids with packaging plasmids pMD2.G and psPAX2. Culture supernatants were collected 48 h after transfection and were used to infect NB4 cells and cells isolated from APL bone marrow samples in the presence of 8 μg/ml of polybrene (Sigma-Aldrich, St. Louis, MO, USA). Expression levels of targeted genes were detected by qRT-PCR or western blot. The sequences of shRNAs targeting KCNQ1OT1 were sh1, 5′-GCCAATGGATAGAGAGCAA-3′; sh2, 5′-GCCAATAGCAACTGACTAA-3′; sh3, 5′-GCCACATCTAACACCTATA-3′; sh4, 5′-GGTGAGAAACCTCTAACAA-3′. The RNAi Consortium (TRC) human genome-wide shRNA collection was used to make gene knockdown cells. MAP3K1 targeting shRNA [19] was TRCN0000197225. FUS targeting shRNA [20] was TRCN0000039824. c-Myc targeting shRNA [21] was TRCN0000039642. 5′-AGCGUGUAGCUAGCAGAGG-3′ was used as negative control sequence.

### Retroviral construct and transfection

The sequence encoding full-length MAP3K1 was amplified from cDNA of NB4 cells and then directionally cloned into retroviral vector MigR1, by Xho I and EcoR I sites, to form plasmid MigR1-MAP3K1. Retroviral particles were produced by co-transfecting 293T cells with packaging plasmids VSV-G and gag-pol. The following procedure was the same as lentivirus production.

### Plasmid constructions and site-directed mutagenesis

A 2462 bp DNA fragment encompassing the MAP3K1 transcription start site and a 568 bp KCNQ1OT1 promoter fragment were respectively amplified by PCR using genomic DNA from NB4 cells. The PCR products were cloned into the pGL3-basic reporter plasmid (Promega, Madison, WI, USA). Mutations of the predicted c-Myc binding sites in the pGL3-KCNQ1OT1 construct were made using the QuikChange site-directed mutagenesis kit (Stratagene, La Jolla, CA, USA) following the manufacturer’s protocol. The c-Myc sequence was amplified using NB4 cDNA and then cloned into the pcDNA3.1 (+) vector. Detailed primer information is listed in Table S2.

### Transient transfection and luciferase reporter assay

NB4 cells were electro-transfected using the Amaxa Nucleofector II device (Lonza, Cologne, Germany) with Nucleofector Kit V (Lonza); 293T cells were transfected with Lipofectamine 2000 (Invitrogen) according to the manufacturer’s instructions. The detailed procedure was described previously [22]. Renilla luciferase plasmid pRL-SV40 acted as an internal control to normalize transfection efficiencies. Firefly and Renilla luciferase activities were measured using the Dual-Luciferase Reporter Assay System reagents (Promega) 24 h after transfection.

### RNA immunoprecipitation (RIP) assay

RIP was performed using the EZ-Magna RIP RNA-Binding Protein Immunoprecipitation Kit (Millipore, Billerica, MA, USA) following the manufacturer’s instructions. Immunoprecipitated RNA was subjected to qRT-PCR analysis to detect MAP3K1 and KCNQ1OT1 expression.

### RNA pulldown assay

MAP3K1 mRNA 3′-UTR was in vitro transcribed by using T7 RNA polymerase (NEB, Ipswich, MA, USA), purified with the RNeasy Plus Mini Kit (Qiagen, Hilden, Germany), and treated with RNase-free DNase I (Qiagen). Then transcribed MAP3K1 mRNA 3′-UTR was labeled with biotin using the Biotin RNA Labeling Mix (Sigma). The biotinylated MAP3K1 3′-UTR was incubated with protein extract obtained from NB4 cells. After that, streptavidin magnetic beads were used to isolate the RNA-protein complex. Finally, the complex was analyzed by western blot.

### Statistical analysis

The data were analyzed statistically using the Student’s *t*-test. Values were the mean ± standard error of the mean (S.E.M.) obtained from at least three independent experiments. *P* value of less than 0.05 was considered as statistical significance. * indicates *p* < 0.05, ** indicates *p* < 0.01, *** indicates *p* < 0.001, **** indicates *p* < 0.0001.

## Results

### KCNQ1OT1 is significantly highly expressed in APL

**Fig. 1:**
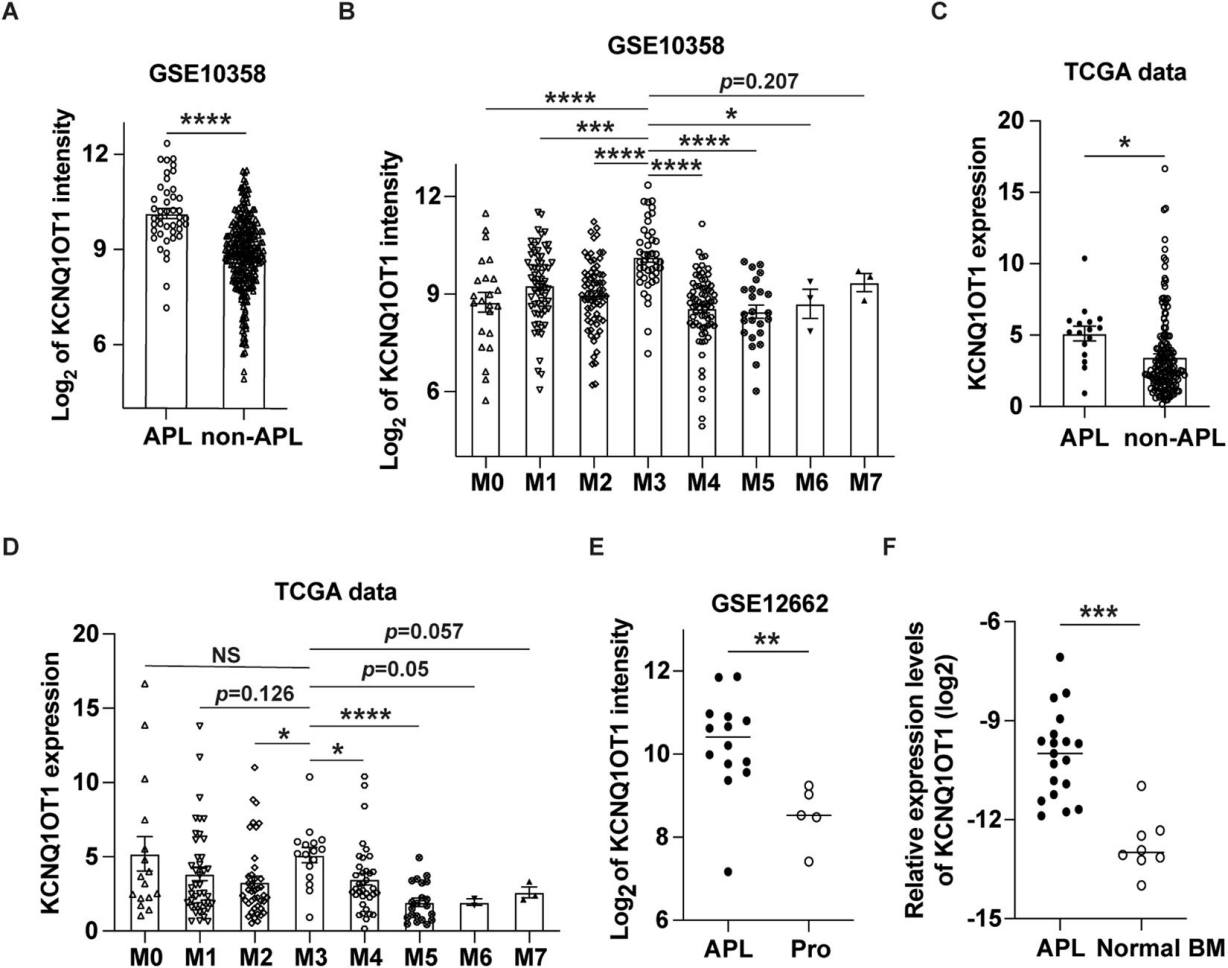
KCNQ1OT1 is highly expressed in APL. **A**–**D** The expression of KCNQ1OT1 in different FAB subtypes from two AML patient cohorts (GSE10358 and TCGA). **E** The expression of KCNQ1OT1 in APL and normal promyelocytes (Pro) was retrieved from GEO (GSE12662). **F** KCNQ1OT1 expression was determined in 19 primary APL patient samples and eight normal bone marrows (Normal BM). The result is presented with the means ± SEM. *p < 0.05, **p < 0.01, ***p < 0.001, ****p < 0.0001. NS not significant.

First, we analyzed the expression level of KCNQ1OT1 with GEPIA [23], a web-based tool to compare gene expression based on TCGA [24] and GTEx data [25]. Among all tumor samples and paired normal tissues, KCNQ1OT1 was exclusively highly expressed in AML (Fig. S1). Then we downloaded and analyzed the human AML gene expression microarray profile (GSE10358) from the GEO database, and found that the expression of KCNQ1OT1 was especially higher in APL (AML-M3) than other AML subtypes (Fig. 1A, B). The results were confirmed by the data downloaded from the TCGA database (Fig. 1C, D). Subsequently, the expression of KCNQ1OT1 in APL and normal promyelocytes was retrieved from GEO (GSE12662). As a result, KCNQ1OT1 was indeed upregulated in APL compared with normal promyelocytes (Fig. 1E). To verify the microarray results, 19 primary APL patient samples and eight normal bone marrows were collected and expression of KCNQ1OT1 was examined (Fig. 1F). Collectively, these results suggest that KCNQ1OT1 is selectively highly expressed in APL.

### Upregulated KCNQ1OT1 promotes APL cell proliferation

**Fig. 2:**
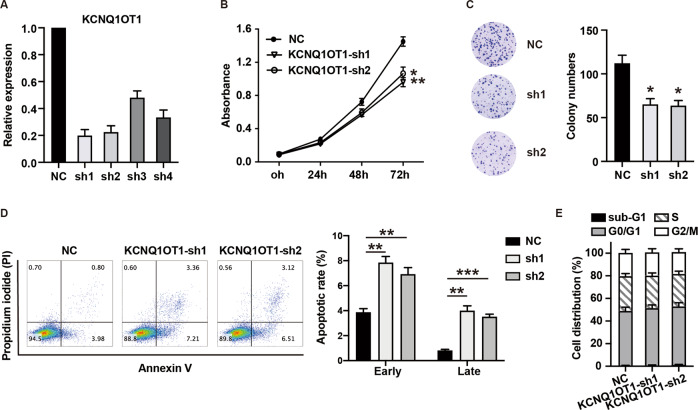
Highly expressed KCNQ1OT1 promotes APL cell proliferation. **A** Four shRNAs were used to silence KCNQ1OT1 in NB4 cells. The expression of KCNQ1OT1 was determined in NB4 cells transfected with pLVX-shRNA2-derived lentiviruses carrying shRNAs specifically targeting KCNQ1OT1. **B** CCK8 assays were performed to determine the viability of NB4 cells transfected with KCNQ1OT1-sh1, KCNQ1OT1-sh2, and negative control (NC). **C** Colony formation assays were performed to test the proliferation of KCNQ1OT1-shRNA-transfected NB4 cells. Colonies were captured and counted. **D** Cell apoptosis was analyzed by flow cytometry. Lower Right: early apoptotic cells (Early), Upper Right: late (or terminal) apoptotic cells (Late). **E** Cell cycle distribution was determined by flow cytometry. The result is presented with the means ± SEM of three independent experiments. *p < 0.05, **p < 0.01, ***p < 0.001.

To explore the biological function of upregulated KCNQ1OT1 in APL, four shRNAs were used to silence KCNQ1OT1 in APL patient-derived NB4 cells. As shown in Fig. 2A, KCNQ1OT1-sh1 (sh1) and KCNQ1OT1-sh2 (sh2) silenced KCNQ1OT1 more efficiently, so they were selected for the following experiments. Cell proliferation assays (CCK8) showed that knockdown of KCNQ1OT1 significantly reduced cell growth (Fig. 2B). Consistently, silencing of KCNQ1OT1 also markedly decreased the colony formation ability of NB4 cells (Fig. 2C). To explore whether KCNQ1OT1 is involved in cell apoptosis and cell cycle progression, flow cytometry analysis was performed. Silencing of KCNQ1OT1 increased the apoptotic rate of NB4 cells (Fig. 2D). However, knockdown of KCNQ1OT1 had no obvious effect on cell cycle progression (Fig. 2E). These results demonstrated that upregulated KCNQ1OT1 promoted APL cell proliferation whereas inhibited cell apoptosis.

### KCNQ1OT1 promotes APL cell proliferation by regulating MAP3K1

**Fig. 3:**
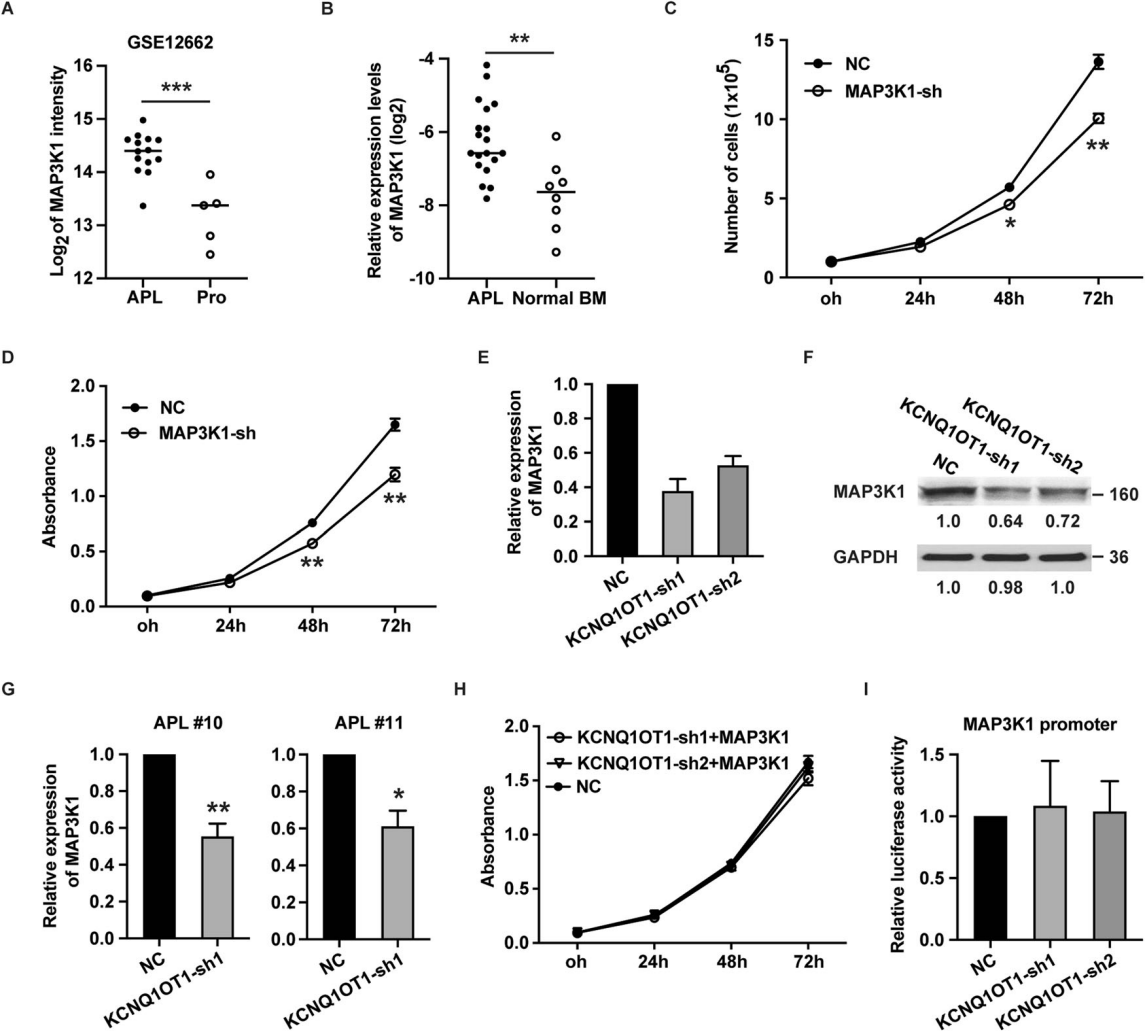
KCNQ1OT1 positively regulates MAP3K1. **A** The expression of MAP3K1 in APL and normal promyelocytes was retrieved from GEO (GSE12662). **B** The expression of MAP3K1 was detected in 19 primary APL patient samples and eight normal bone marrow samples. **C**, **D** Cell proliferation was determined by cell count and CCK8 assay in NB4 cells infected with MAP3K1-shRNA at indicated time points. **E**, **F** The RNA and protein level of MAP3K1 was determined in NB4 cells infected with KCNQ1OT1-sh1 and KCNQ1OT1-sh2. **G** The expression of MAP3K1 was detected in two primary APL BM samples infected with KCNQ1OT1-sh1 by qRT-PCR. **H** MAP3K1 was overexpressed in NB4 cells with silencing of KCNQ1OT1. Cell proliferation was determined by CCK8 assay at indicated time points. **I** pGL3-MAP3K1 promoter luciferase plasmid was transfected into NB4 cells that stably expressing KCNQ1OT1-sh1 and KCNQ1OT1-sh2. Luciferase activity was measured 24 h after transfection. The result is presented with the means ± SEM of three independent experiments. *p < 0.05, **p < 0.01, ***p < 0.001.

To investigate the potential downstream targets of KCNQ1OT1, the GEPIA database was browsed and MAP3K1, a crucial member of the MAPK signaling cascade, was found to be highly expressed in AML (Fig. S2). Importantly, there was a significant positive correlation between MAP3K1 and KCNQ1OT1 in AML (Fig. S3). Then, the expression of MAP3K1 in APL and normal promyelocytes was retrieved from GEO (GSE12662). As shown in Fig. 3A, the expression level of MAP3K1 was higher in APL than that in normal promyelocytes. The results were validated by our clinical cohort (Fig. 3B). To elucidate the role of MAP3K1 in APL cell proliferation, shRNA specifically targeting MAP3K1 was used to silence MAP3K1 (Fig. S4). As shown in Fig. 3C, 3D, knockdown of MAP3K1 drastically reduced NB4 cell proliferation. Thereafter, we aimed to clarify the association between KCNQ1OT1 and MAP3K1. Our results revealed that KCNQ1OT1 silencing obviously diminished the expression of MAP3K1 both at mRNA and protein levels (Fig. 3E, F), indicating that KCNQ1OT1 could regulate the expression of MAP3K1. The findings were validated in two APL bone marrow samples (Fig. 3G). Subsequently, a rescue assay was used to elucidate the role of MAP3K1 in the KCNQ1OT1-mediated proliferation of APL cells. Reduced cell growth in NB4 cells with KCNQ1OT1 silencing was reversed by overexpression of MAP3K1 (Fig. 3H). All the above results suggest that KCNQ1OT1 promotes APL cell proliferation by regulating MAP3K1. To explore the regulatory mechanism of KCNQ1OT1 on MAP3K1, we examined whether KCNQ1OT1 could transactivate the promoter of MAP3K1. Luciferase reporter assays demonstrated that MAP3K1 promoter activity was unaffected by silencing of KCNQ1OT1 (Fig. 3I), suggesting that KCNQ1OT1 might modulate MAP3K1 expression at the post-transcriptional level.

### FUS binds to and stabilizes MAP3K1 mRNA in APL

**Fig. 4:**
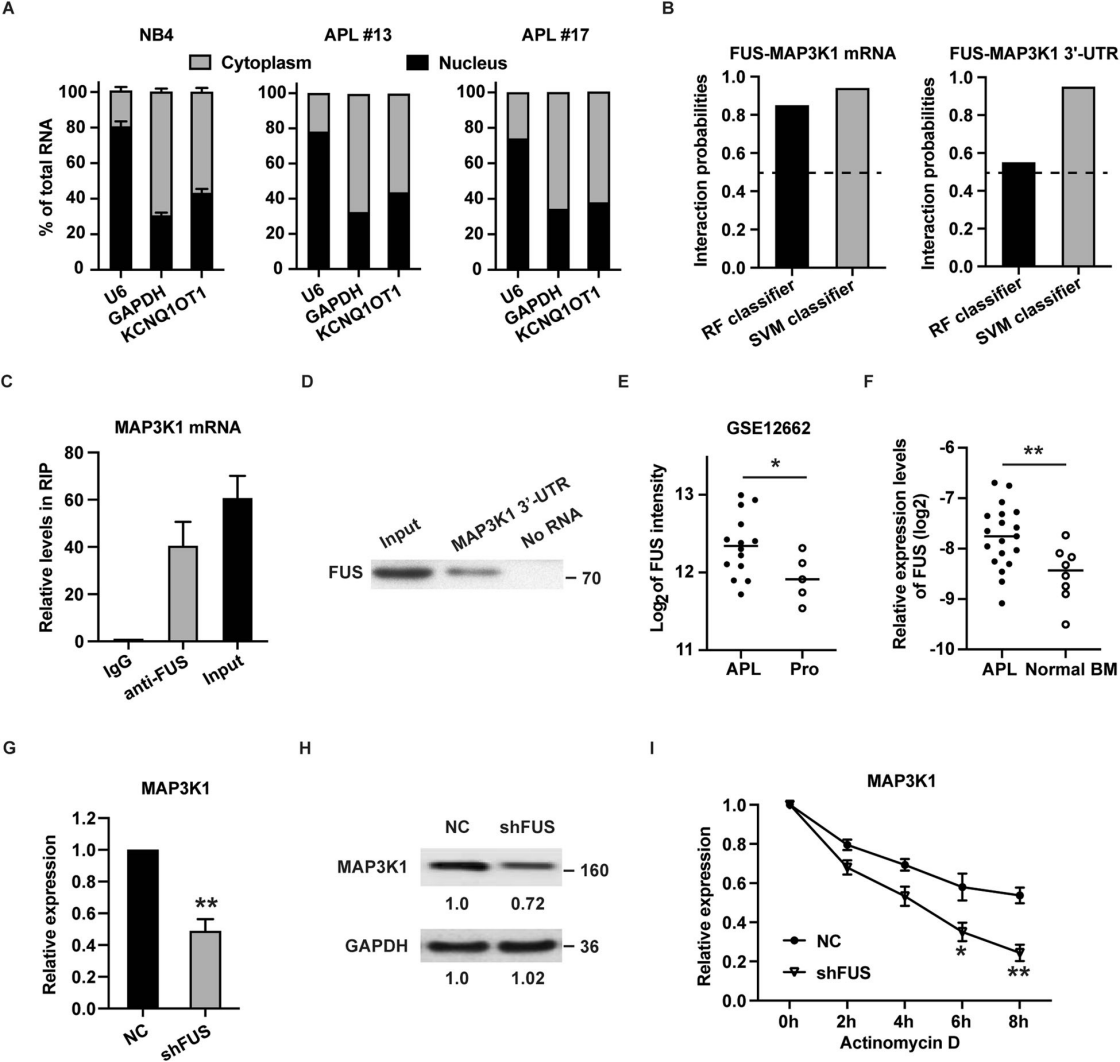
FUS stabilizes MAP3K1 mRNA in APL cells. **A** The expression levels of KCNQ1OT1 in the cytoplasm and nucleus of NB4 cells and two APL patient samples were detected by qRT-PCR. U6 was used as a nucleus marker and GAPDH was used as a cytosol marker. **B** The interaction probabilities of FUS with MAP3K1 mRNA and 3′-UTR of MAP3K1 mRNA were predicted by the RNA-Protein interaction prediction (RPISeq) website. Predictions with probabilities >0.5 were considered “positive”, indicating that the corresponding RNA and protein are likely to interact. **C** RIP experiments were performed in NB4 cells, and the coprecipitated RNA was subjected to qRT-PCR for MAP3K1 expression. The fold enrichment of MAP3K1 mRNA in FUS RIP is relative to the IgG control. **D** The biotinylated MAP3K1 3′-UTR was used to pull down FUS. Western blot assay was used to detect the FUS in MAP3K1 3′-UTR precipitates. **E** The expression of FUS in APL and normal promyelocytes was retrieved from GEO (GSE12662). **F** The expression level of FUS was determined in 19 primary APL patient samples and eight normal bone marrow samples by qRT-PCR. **G**, **H** The RNA and protein level of MAP3K1 was detected in NB4 cells transfected with shRNA specifically targeting FUS (shFUS). **I** RNA stability assays were performed using Actinomycin D to disrupt RNA synthesis in NB4 cells, and the expression of MAP3K1 mRNA was measured every 2 h. The result is presented with the means ± SEM of three independent experiments. *p < 0.05, **p < 0.01.

To explore the potential mechanism of KCNQ1OT1 in APL, subcellular localization was detected. The level of KCNQ1OT1 was higher in the cytosol than that in the nucleus in both NB4 cells and APL patient samples (Fig. 4A). Cytoplasmic lncRNAs are well-known to interact with RNA binding proteins (RBPs) [26]. From the ENCORI database (http://starbase.sysu.edu.cn/index.php), FUS was predicted as a shared RBP for KCNQ1OT1 and MAP3K1 mRNA (Table S3 and S4). Then the interaction between FUS and MAP3K1 mRNA was further predicted by the RNA–Protein interaction prediction (RPISeq) website. The scores of RF classifier and SVM classifier were both over 0.5 (Fig. 4B), suggesting that FUS had a great possibility of interaction with MAP3K1 mRNA. Because RBP interactions with 3′-UTR are important determinants of mRNA stability [27], and FUS tend to bind 3′-UTR of MAP3K1 mRNA (Fig. 4B), the 3′-UTR was used to perform RNA pulldown. RNA immunoprecipitation (RIP) and RNA pulldown assay results showed that FUS highly bound to MAP3K1 mRNA (Fig. 4C, D).

Furthermore, by browsing GEPIA website, we found that FUS was positively correlated with MAP3K1 in AML (Fig. S5), indicating that FUS may positively regulate MAP3K1 mRNA. We retrieved the gene expression data from GEO (GSE12662) and found that, similar to MAP3K1, FUS expression was also higher in APL than normal promyelocytes (Fig. 4E). The result was confirmed by our clinical samples (Fig. 4F). To test the influence of FUS on MAP3K1, level of MAP3K1 was determined in NB4 cells transfected with shRNA targeting FUS. As revealed in Fig. 4G, H, knockdown of FUS significantly downregulated MAP3K1 both at mRNA and protein levels. Because FUS is known to act as an mRNA stabilizer [28], we further explored whether FUS could modulate MAP3K1 mRNA stability. As expected, after treatment with Actinomycin D, the half-life of MAP3K1 mRNA was decreased by silencing FUS (Fig. 4I). To sum up, FUS binds to and stabilizes MAP3K1 mRNA in APL cells.

### KCNQ1OT1 and FUS synergistically stabilize MAP3K1 mRNA

**Fig. 5:**
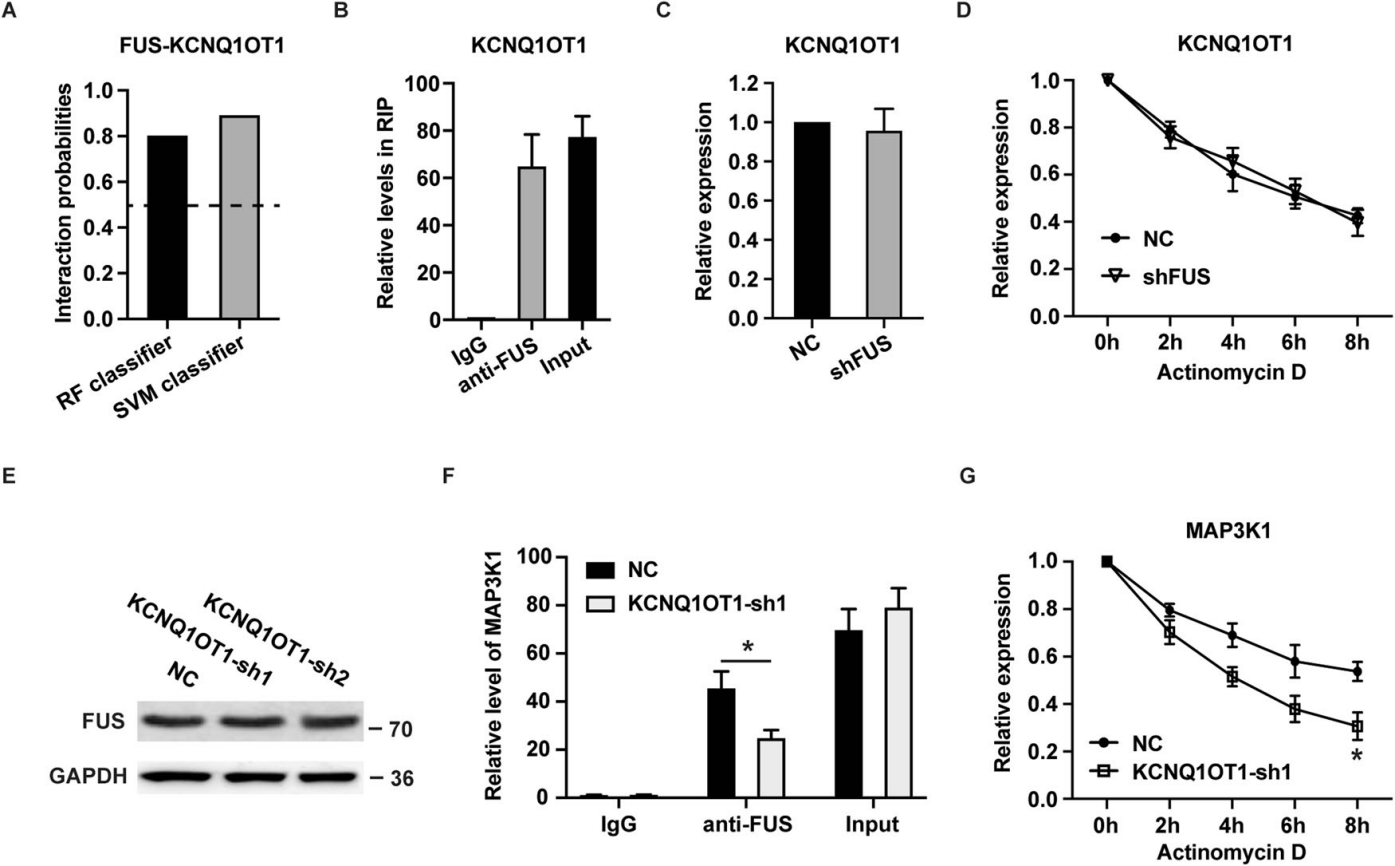
KCNQ1OT1 and FUS positively regulate MAP3K1 mRNA stability. **A** The interaction probability between FUS and KCNQ1OT1 was predicted by the RPISeq website. **B** The fold enrichment of KCNQ1OT1 was analyzed in FUS RIP in NB4 cells. **C** The expression of KCNQ1OT1 was analyzed in NB4 cells transfected with shFUS. **D** The expression level of KCNQ1OT1 in NB4 cells infected with shFUS and negative control were analyzed every 2 h after treatment with actinomycin D. **E** Western blot assay was performed to determine the expression level of FUS in NB4 cells infected with KCNQ1OT1-sh1 and KCNQ1OT1-sh2. **F** The fold enrichment of MAP3K1 was determined in FUS RIP in NB4 cells infected with KCNQ1OT1-sh1 and negative control (NC). **G** The level of MAP3K1 mRNA in NB4 cells infected with KCNQ1OT1-sh1 and negative control was analyzed every 2 h after treatment with actinomycin D. The data are shown with the means ± SEM of three independent experiments. *p < 0.05.

Next, the RPISeq website was used to further predict the interaction between KCNQ1OT1 and FUS. The scores of RF classifier and SVM classifier were 0.8 and 0.89, respectively (Fig. 5A), indicating that FUS has a good chance of binding to KCNQ1OT1. The result was validated by RIP assays in NB4 cells (Fig. 5B). However, silencing FUS did not exert any influence on KCNQ1OT1 expression (Fig. 5C) and KCNQ1OT1 stability (Fig. 5D). Similarly, the level of FUS was unchanged in NB4 cells with downregulated KCNQ1OT1 (Fig. 5E). These results together indicated that FUS does not affect KCNQ1OT1 expression. Importantly, knockdown of KCNQ1OT1 markedly decreased the interaction between FUS and MAP3K1 mRNA in NB4 cells (Fig. 5F). Consistently, silencing of KCNQ1OT1 in NB4 cells obviously reduced the stability of MAP3K1 mRNA (Fig. 5G). Taken together, our results suggest that KCNQ1OT1 and FUS synergistically mediate MAP3K1 mRNA stability.

### c-Myc transactivates KCNQ1OT1 expression in APL

**Fig. 6:**
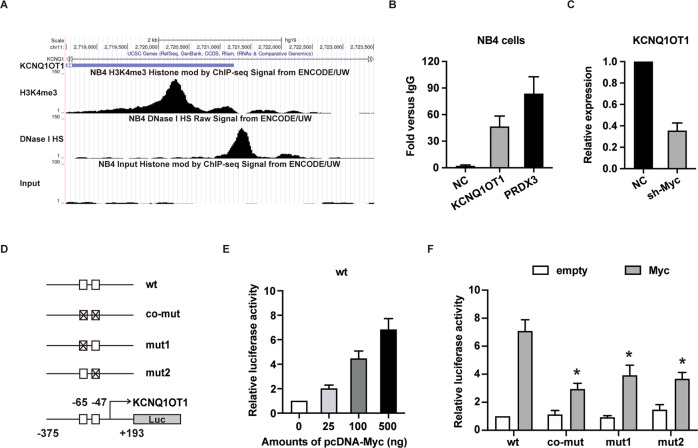
c-Myc transactivates KCNQ1OT1 expression in APL cells. **A** Genome browser screenshot of the KCNQ1OT1 locus. H3K4Me3 (GSM945275), DNase I HS signal (GSM736604), and Input (GSM945252) ChIP-seq signals of NB4 cells were displayed in the UCSC genome browser. **B** ChIP assay was performed to investigate the interaction between c-Myc and KCNQ1OT1 promoter. PRDX3 was used as the positive control. **C** The expression level of KCNQ1OT1 was determined in NB4 cells infected with shRNA specifically targeting c-Myc (shMyc). **D** Schema of the KCNQ1OT1 promoter region shows the different mutation constructs used in this study. □ represents the wild-type c-Myc binding site and ⊠ represents the mutated site. **E** The 568 bp KCNQ1OT1 promoter reporter construct was transfected into 293T cells along with increasing amounts of the pcDNA-Myc expression plasmid. Luciferase activity was measured 24 h after transfection. **F** The luciferase promoter constructs with mutated −65 and/or −47 sites were co-transfected with 500 ng of the pcDNA3.1 (empty) or pcDNA-Myc (Myc) plasmid into 293T cells. Luciferase activities were measured 24 h after transfection. The result is presented with the means ± SEM of at least three independent experiments. *p < 0.05.

DNase I hypersensitive site (DNase I HS) and enrichment of H3K4me3 have been combined to mark active promoters [29–31]. To interrogate the mechanism of KCNQ1OT1 upregulation in APL, we utilized the DNase I HS-sequencing and chromatin immunoprecipitation-sequencing (ChIP-seq) data of NB4 cells generated by the ENCODE Project Consortium [32] to identify the regulatory region of KCNQ1OT1. The results were displayed using UCSC Genome Browser (http://genome.ucsc.edu) [33]. As shown in Fig. 6A, DNase I HS was found to be located around the transcription start site (TSS), and the enrichment of H3K4me3 binding was located around the TSS and around +1000 bp downstream of TSS. These results suggested that the region from −200 bp to +1200 bp of TSS might mediate the transcription of KCNQ1OT1. c-Myc, a crucial transcription factor involved in proliferation and leukemogenesis, is upregualted in APL [34]. Therefore, c-Myc binding sites on KCNQ1OT1 promoter were predicted with the JASPAR tool. The results revealed two significantly enriched binding sites for c-Myc around the TSS (Fig. S6 and Fig. 6D). Next, ChIP-qPCR was used to detect the binding of c-Myc on the KCNQ1OT1 promoter. The results demonstrated that c-Myc bound to KCNQ1OT1 promoter (Fig. 6B). To clarify the role of c-Myc in KCNQ1OT1 activation, shRNA specifically targeting c-Myc was introduced into NB4 cells. As shown in Fig. 6C, silencing of c-Myc drastically decreased the expression of KCNQ1OT1 in NB4 cells. Thereafter, promoter-reporter assays were conducted in 293T cells. The KCNQ1OT1 promoter construct containing potential c-Myc binding sites was co-transfected with increasing amounts of the c-Myc expression construct. As shown in Fig. 6E, the KCNQ1OT1 promoter was transactivated by c-Myc in a dose-dependent manner. On the contrary, mutation of either one or both c-Myc binding sites (Fig. 6D) significantly diminished the c-Myc-mediated transactivation (Fig. 6F). Cumulatively, our results demonstrated that c-Myc bound to and transactivated KCNQ1OT1 promoter in APL.

## Discussion

KCNQ1OT1 has been reported to be involved in lung cancer [13, 15], tongue cancer [35], glioma [16], colorectal cancer [17], bladder cancer [36], prostate cancer [12], and AML [37, 38]. In AML, KCNQ1OT1 contributes to AML cell proliferation through regulating c-Myc and Tspan3 via ceRNA mechanism in U937, HL-60, and K562 cells [37, 38]. However, the expression and function of KCNQ1OT1 in APL are still unknown. Here, we first identified that KCNQ1OT1 was exclusively highly expressed in AML among all tumor samples and paired normal tissues, whereas within AML, expression of KCNQ1OT1 was higher in APL than other subtypes of AML. Notably, upregulated KCNQ1OT1 promoted the proliferation of APL patient-derived NB4 cells.

KCNQ1OT1 was primarily identified as a chromatin regulatory RNA through binding to histone methyltransferases G9a and the PRC2 complex and DNA methyltransferase 1 (DNMT1) [8]. Recently, KCNQ1OT1 was found to act as a ceRNA through interacting with RNA binding protein AGO2 [16, 17, 37, 38]. These findings raise the possibility that KCNQ1OT1 could interact with other RNA binding proteins. However, the interaction between KCNQ1OT1 and RNA binding proteins is rarely reported. On the other hand, as a well-known RNA binding protein, FUS, is a crucial mRNA stabilizer in cytoplasm. For instance, FUS interacts with lncRNA CTBP1-AS2 to stabilize TLR4 in cardiomyocyte hypertrophy [39]. FUS could also bind to lncRNA DLX6-AS1 to regulate MAP4K1, thus promoting cell proliferation, migration and EMT of gastric cancer [40]. In this study, we demonstrated that KCNQ1OT1 was mainly located in the cytoplasm of NB4 cells and APL patient samples. Cytoplasmic KCNQ1OT1 bound to FUS, and KCNQ1OT1/FUS complex synergistically stabilized MAP3K1 mRNA, thus facilitating APL cell proliferation. To the best of our knowledge, our study is the first to report that KCNQ1OT1 interacts with RBP FUS to stabilize downstream targets, thus contributing to the proliferation of APL cells. Our findings not only reveal a previously unidentified role of KCNQ1OT1 in facilitating APL cell proliferation, but also uncover the molecular mechanism of how KCNQ1OT1 participates in APL development.

As a proto-oncogenic transcription factor, c-Myc plays a vital role in cell proliferation, cell transformation and tumorigenesis [41, 42]. Clinically, c-Myc is overexpressed or amplified among various human cancers, including AML [43–45]. A less than 2-fold increase in c-Myc levels can transform monocyte-macrophages [46]. In mice, transduction of myeloid cells with c-Myc causes AML development [47]. Though c-Myc plays an essential role in leukemogenesis, known targets of c-Myc in AML are limited. It is reported that c-Myc-bound promoters are always with an active chromatin state characterized by specific histone marks, such as H3K4me3 and hypersensitivity to digestion by DNase I [48]. In this study, we used the published data of DNase I HS-seq and H3K4me3 ChIP-Seq to predict the active promoter of KCNQ1OT1 in NB4 cells, and then found that c-Myc bound to and transactivated the KCNQ1OT1 promoter. Our results describe an important role of KCNQ1OT1 in mediating c-Myc-induced leukemia cell proliferation and enrich the mechanism of how c-Myc contributes to APL pathogenesis.

The mitogen-activated protein kinase (MAPK) pathway plays a critical role in APL cell proliferation [49, 50]. MAP3K1 is a serine/threonine kinase and is a pivotal upstream regulator in MAPK signaling. In vivo mutagenesis screening has identified MAP3K1 as a driver in the development of melanoma in mice [51]. In addition, copy number loss and somatic mutations of MAP3K1 were reported in a significant fraction of human tumor samples. The resultant impairment of MAP3K1 led to defects in pro-survival signaling, which reduced tumor growth and metastasis [52]. Consistently, MAP3K1 mutations were frequently observed in luminal A subtype of breast cancer. These patients were characterized by better overall survival and lower relapse rates than other subtypes [53]. Above results suggest that MAP3K1 is involved in cancer development. Interestingly, we found that lncRNA KCNQ1OT1/FUS complex stabilized MAP3K1 and upregulated MAP3K1 contributes to APL cell proliferation. Our results indicate that MAP3K1 may also function as a downstream effector of other modulators, including lncRNA, to play a role in cancer cell proliferation.

It would be interesting to identify the exact binding sites of FUS on KCNQ1OT1. However, KCNQ1OT1 is a 91 kb-long transcript and there are hundreds of potential FUS binding sites on KCNQ1OT1 predicted by ENCORI and RBPmap websites. Besides, interactions between lncRNAs and RNA binding proteins may be influenced by the secondary and/or tertiary structure of lncRNAs [54]. We will try to resolve this issue in future work. In addition, we observed that silencing of KCNQ1OT1 increased the apoptosis of APL cells. We will clarify the mechanism of how KCNQ1OT1 affects APL cell apoptosis in the following study. Another limitation of this work is, the experiments to clarify the function and mechanism of KCNQ1OT1 were mainly performed in vitro, more in vivo studies are needed to further verify the function of KCNQ1OT1 in APL.

**Fig. 7:**
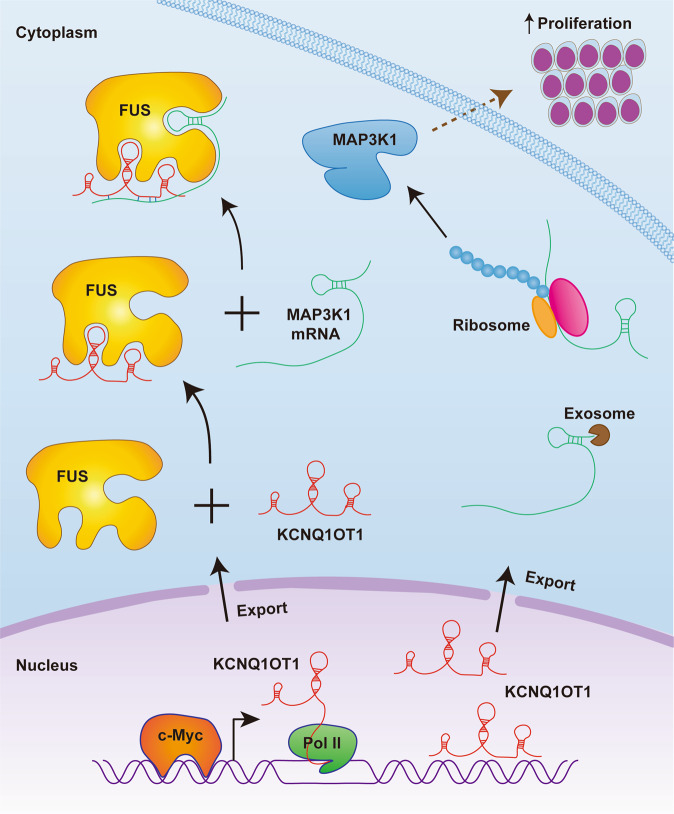
Schematic diagram of lncRNA-KCNQ1OT1 functions to promote proliferation in APL. Light blue background shows cytoplasm, light pink background shows nucleus. Pol II: RNA polymerase II.

In sum, the present study not only revealed that c-Myc transactivated KCNQ1OT1 in APL and upregulated KCNQ1OT1 promoted APL cell proliferation, but also demonstrated that KCNQ1OT1 bound to FUS, thus stabilizing the mRNA of MAP3K1 (Fig. 7). This study established a novel role of the c-Myc/KCNQ1OT1/FUS/MAP3K1 axis in APL cell proliferation and may offer new targets for APL therapy.

## Supplementary information

Supplementary figure and table legends

Figure S1

Figure S2

Figure S3

Figure S4

Figure S5

Figure S6

Table S1

Table S2

Table S3

Table S4

checklist

Author contribution form
