# Supplementary material for: LncRNA KCNQ1OT1 activated by c-Myc promotes cell proliferation via interacting with FUS to stabilize MAP3K1 in acute promyelocytic leukemia
Source: Cell Death Dis. 2021 Aug 17;12(9):795. doi: 10.1038/s41419-021-04080-1 (PMC8371007; doi:10.1038/s41419-021-04080-1)
Supplement: Supplementary file 1 — Supplementary figure and table legends [file 41419_2021_4080_MOESM1_ESM.docx]

**Supplementary figure and table legends**

Figure S1. Expression of KCNQ1OT1 across all tumor samples and paired normal tissues was downloaded from the GEPIA website. KCNQ1OT1 is exclusively highly expressed in AML.

Figure S2. Expression of MAP3K1 among all tumor samples and paired normal tissues was downloaded from the GEPIA website.

Figure S3. Correlation between MAP3K1 and KCNQ1OT1 in AML was analyzed on the GEPIA website.

Figure S4. The expression of MAP3K1 was determined in NB4 cells transfected with shRNA specifically targeting MAP3K1 (MAP3K1-sh) or negative control sequence (NC).

Figure S5. Correlation between MAP3K1 and FUS in AML was analyzed on the GEPIA website.

Figure S6. The c-Myc binding site on KCNQ1OT1 promoter was predicted with the JASPAR tool.

Table S1. Detailed information of patients

Table S2. Primers used for qRT-PCR / construction of plasmids / ChIP-qPCR

Table S3. Predicted RNA binding protein of KCNQ1OT1 by ENCORI

Table S4. Predicted RNA binding protein of MAP3K1 mRNA by ENCORI
