# Supplementary figures and images for: LncRNA KCNQ1OT1 activated by c-Myc promotes cell proliferation via interacting with FUS to stabilize MAP3K1 in acute promyelocytic leukemia
Source: Cell Death Dis. 2021 Aug 17;12(9):795. doi: 10.1038/s41419-021-04080-1 (PMC8371007; doi:10.1038/s41419-021-04080-1)

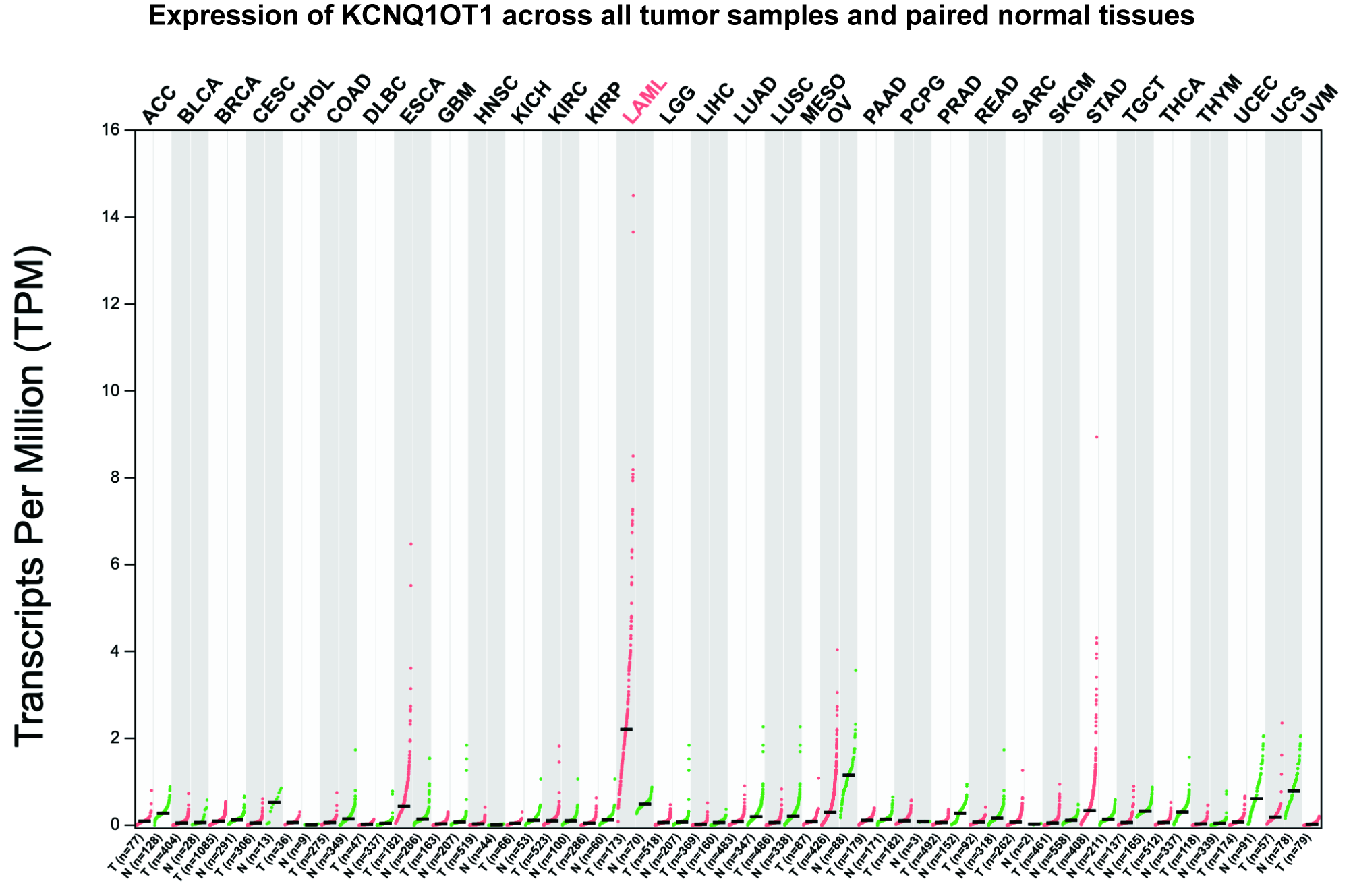

Supplement: Supplementary file 2 — Figure S1 [file 41419_2021_4080_MOESM2_ESM.tif]

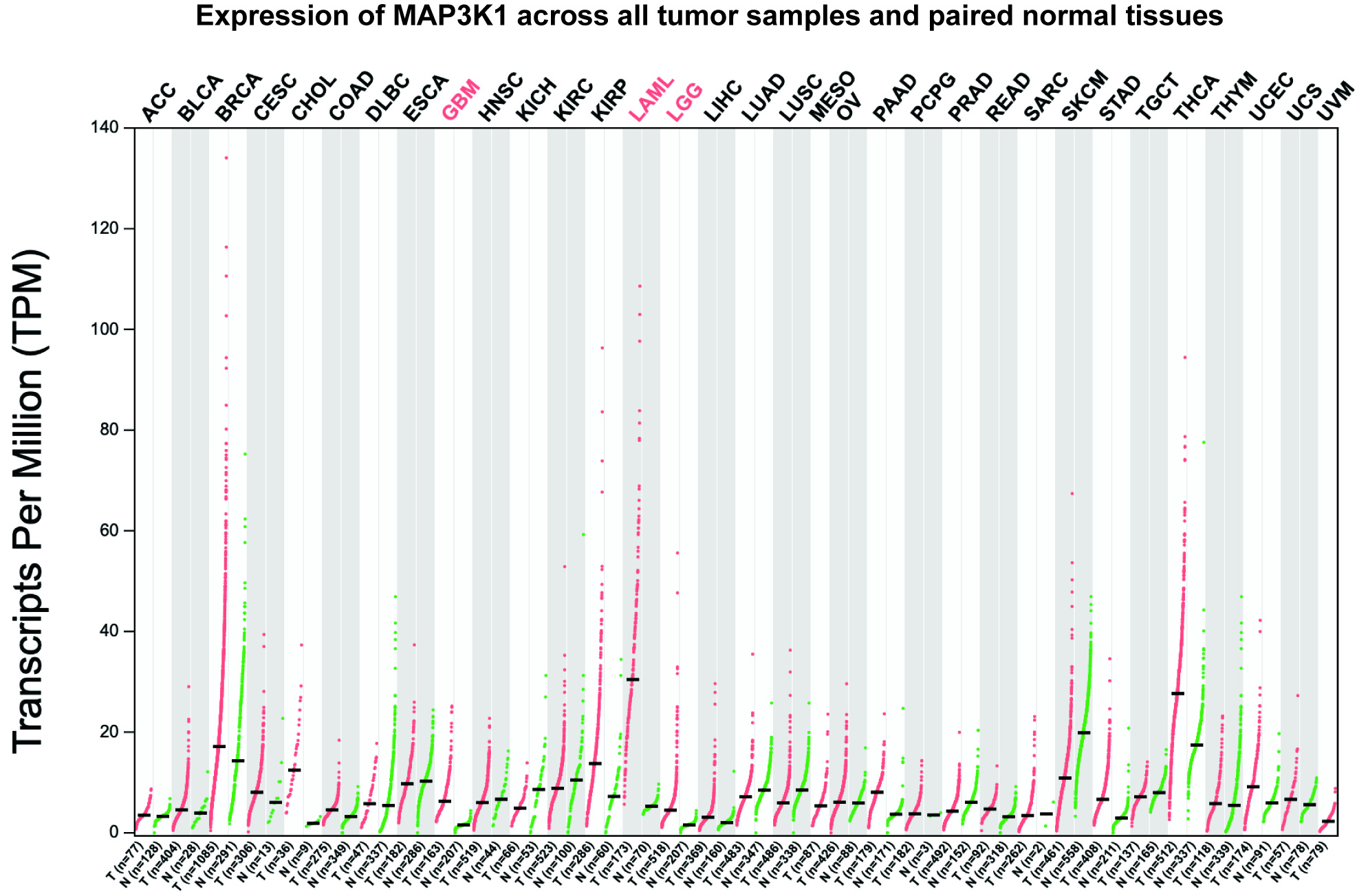

Supplement: Supplementary file 3 — Figure S2 [file 41419_2021_4080_MOESM3_ESM.tif]

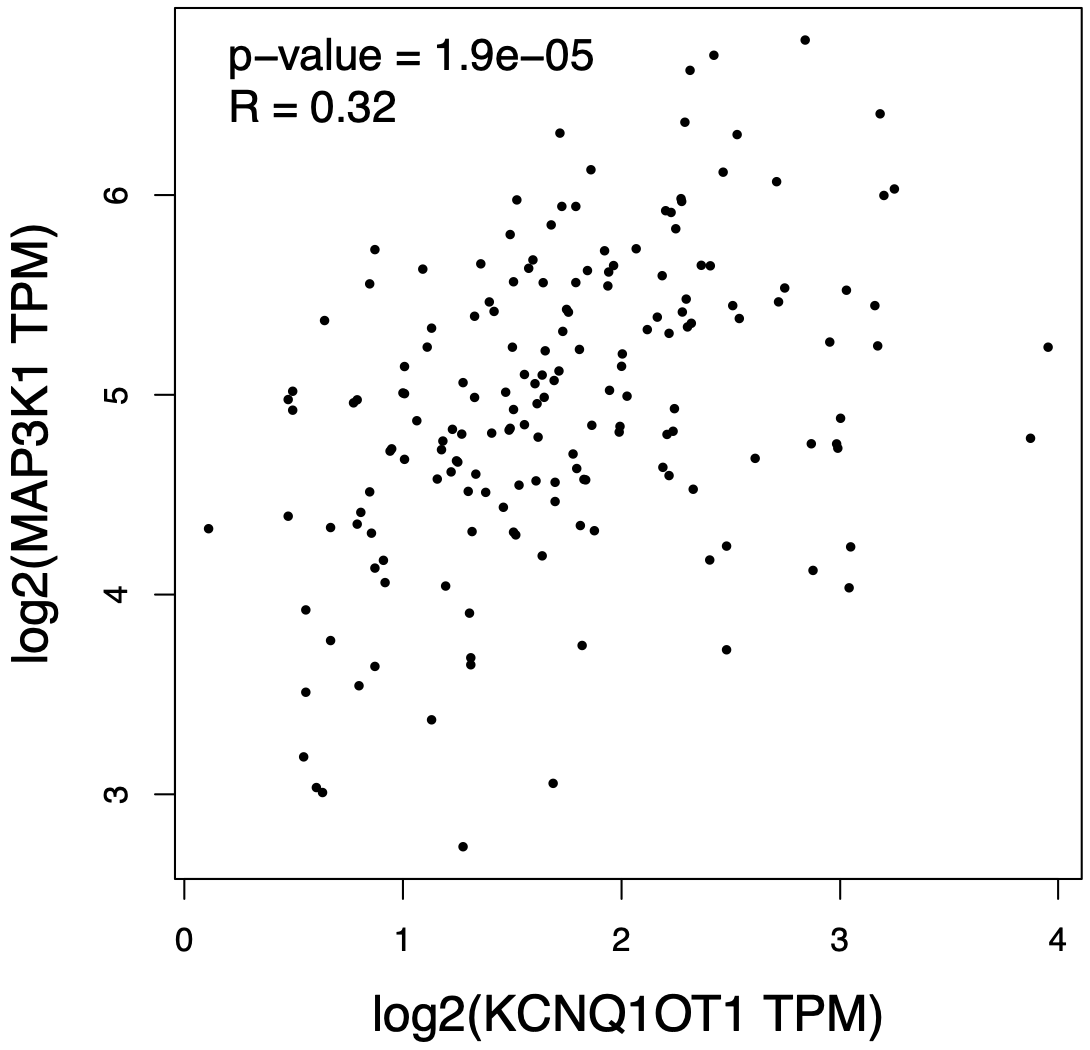

Supplement: Supplementary file 4 — Figure S3 [file 41419_2021_4080_MOESM4_ESM.png]

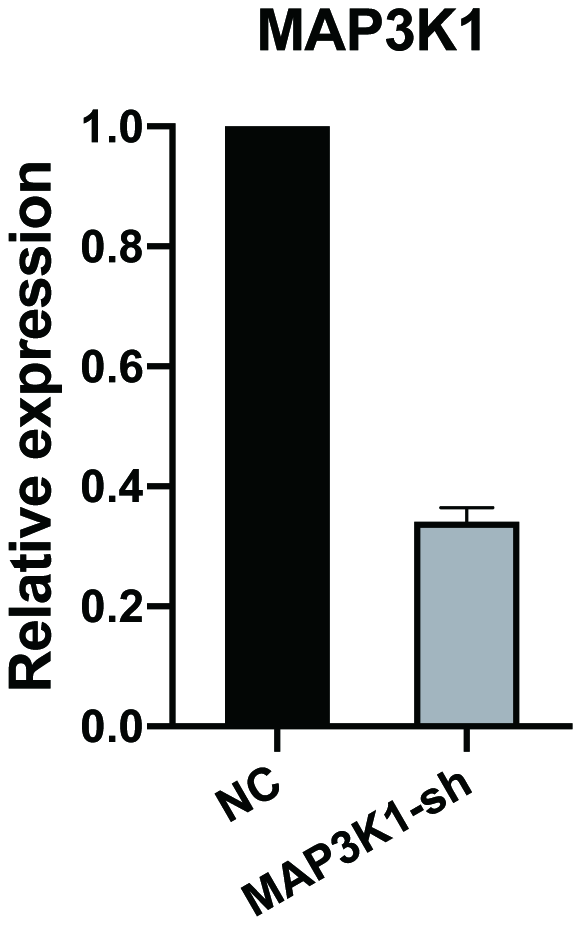

Supplement: Supplementary file 5 — Figure S4 [file 41419_2021_4080_MOESM5_ESM.tif]

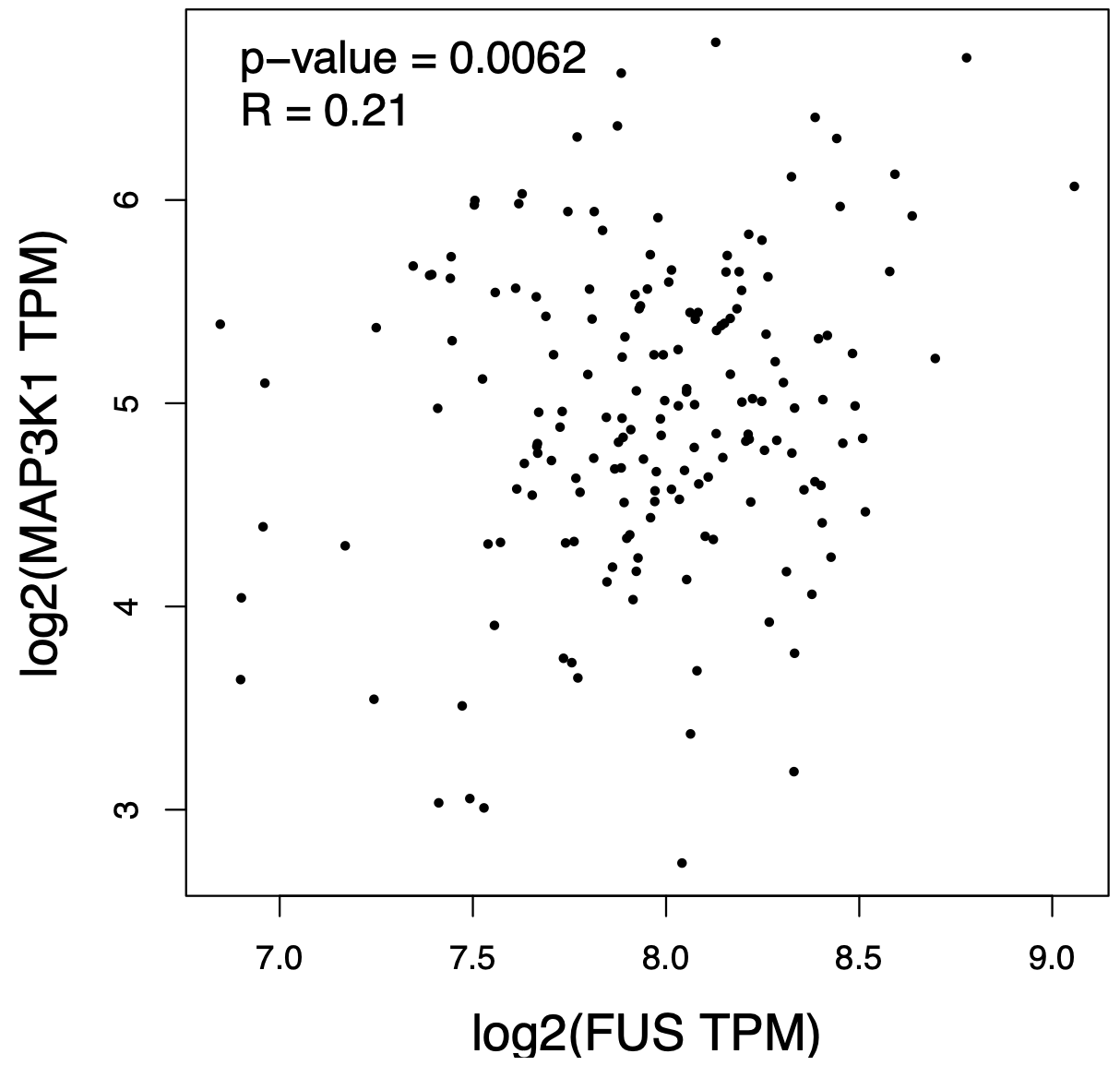

Supplement: Supplementary file 6 — Figure S5 [file 41419_2021_4080_MOESM6_ESM.png]

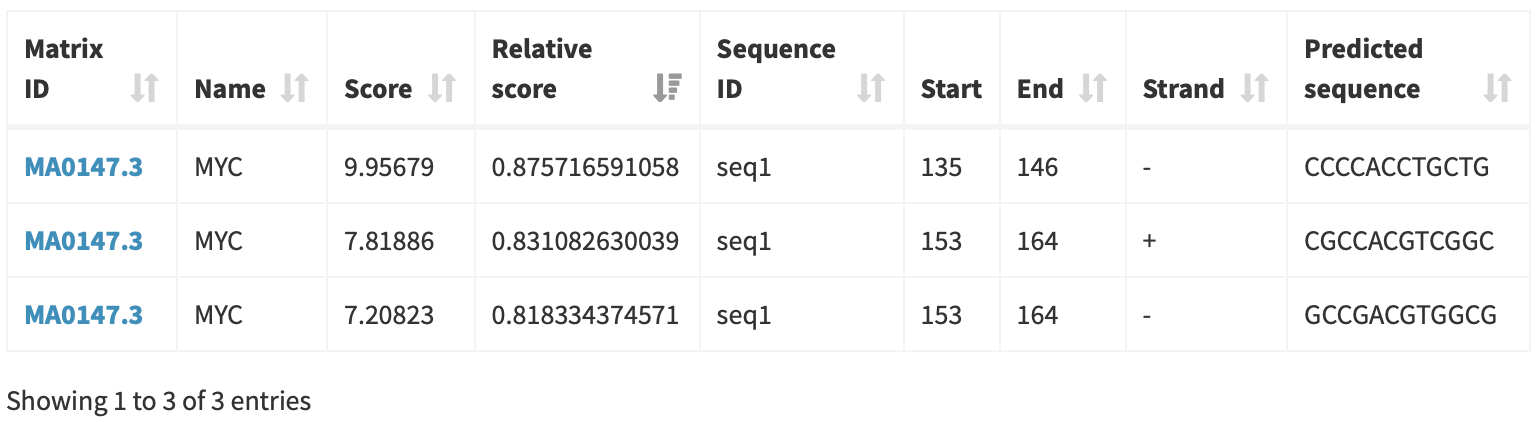

Supplement: Supplementary file 7 — Figure S6 [file 41419_2021_4080_MOESM7_ESM.png]
