# Supplementary material for: LncRNA KCNQ1OT1 activated by c-Myc promotes cell proliferation via interacting with FUS to stabilize MAP3K1 in acute promyelocytic leukemia
Source: Cell Death Dis. 2021 Aug 17;12(9):795. doi: 10.1038/s41419-021-04080-1 (PMC8371007; doi:10.1038/s41419-021-04080-1)
Supplement: Supplementary file 13 — Author contribution form [file 41419_2021_4080_MOESM13_ESM.pdf]

DECLARATION OF CONTRIBUTIONS TO ARTICLE **ADMC**

Manuscript Number:	Journal Name:	
	Cell Death & Disease	(the 'Journal')
Proposed Title of the Contribution:		
LncRNA KCNQ1OT1 activated by c-Myc promotes cell proliferation via interacting with FUS to stabilize MAP3K1 in acute promyelocytic leukemia		(the 'Contribution)
Author(s):		
Doudou Tang, Yujiao Luo, Yafeng Jiang, Piao Hu, Hongling peng, Shangjie Wu, Guangsen Zhang, Yewei Wang		(the 'Authors')

For all *CDDis* articles, each person named as an author in the published version must be able to show he or she has contributed substantially to the article.

Authorship credit should be based on 1) substantial contributions to conception and design, acquisition of data, or analysis and interpretation of data; 2) drafting the article or revising it critically for important intellectual content; and 3) final approval of the version to be published. Authors should meet conditions 1, 2 and 3.

Any person who cannot be shown to have made a substantial contribution to the article cannot be listed as an author in the final version. The name of any person who is deemed to have made a minor contribution can, however, appear in the Acknowledgments section of the article.

Please complete the table below to indicate the contributions of all named authors to the manuscript.

Author Full Name:	Specification of Contribution to the Manuscript:
Doudou Tang	Designed the study, performed experiments, analyzed data, and wrote the manuscript
Yujiao Luo	Designed the study, performed experiments, analyzed data
Yafeng Jiang	Designed the study, performed experiments, analyzed data and revised the manuscript
Piao Hu	Performed experiments, analyzed data
Hongling peng	Analyzed the data and reviewed the manuscript
Shangjie Wu	Analyzed the data and reviewed the manuscript
Guangsen Zhang	Supervised the research and reviewed the manuscript
Yewei Wang	Conceived and designed the study and wrote the manuscript

Please complete the table below to indicate the contributions of all named authors to the figures.

Figure 1:

Fig 1 A,B,C: DT analyzed the data and generated the graph. D: DT and PH collected the patient samples, performed the experiments and generated the data.

Figure 2:

Fig 2 A,B,C: DT generated the data and prepared the graph. D,E: YL generated the data and prepared the graph.

Figure 3:

Fig 3 A-H: DT generated the data and prepared the graph. I: YJ generated the data and prepared the graph.

Figure 4:

Fig 4 A, B: DT generated the data and prepared the graph. C,D: YJ generated the data and prepared the graph. E,F: DT generated the data and prepared the graph. G-I:YL generated the data and prepared the graph.

Figure 5:

Fig 5A: DT generated the data and prepared the graph. B-G: YL generated the data and prepared the graph.

Figure 6:

Fig 6 A,B: YJ generated the data and prepared the graphs. C: DT performed the experiments and generated the data. D-F: YJ performed the experiments and generated the data.

Signed for and on behalf of the Author(s):	Print Name:	Date:
Yewei Wang	Yewei Wang	2021/5/4
